# Inhibition of Mettl3 alleviates low-dose cisplatin-induced renal fibrosis and enhances the chemotherapeutic efficacy in mouse models of cancer

**DOI:** 10.7150/ijbs.117443

**Published:** 2025-06-23

**Authors:** Yuxin Xie, Huiling Li, Jian Pan, Yijian Li, Dongshan Zhang

**Affiliations:** 1 Department of Critical Care Medicine, The Second Xiangya Hospital, Central South University, Changsha, Hunan, People's Republic of China.; 2 Department of Emergency Medicine, The Second Xiangya Hospital, Central South University, Changsha, Hunan, People's Republic of China.; 3 Emergency Medicine and Difficult Diseases Institute, The Second Xiangya Hospital, Central South University, Changsha, Hunan, People's Republic of China.; 4 Department of Ophthalmology, The Second Xiangya Hospital, Central South University, Changsha, Hunan, People's Republic of China.; 5 Department of Urology, The Second Xiangya Hospital, Central South University, Changsha, Hunan, People's Republic of China.; 6 Furong Laboratory, Xiangya School of Medicine, Central South University, Changsha, Hunan, People's Republic of China

**Keywords:** acute kidney injury, renal fibrosis, Mettl3

## Abstract

Cisplatin (CDDP), a commonly utilized anti-tumor drug, leads to acute kidney injury (AKI) and chronic kidney disease (CKD). The mechanisms and therapeutic approaches for injury in AKI have been extensively studied, but the mechanisms resulting in CKD are poorly comprehended and intervention methods are scarce. In the current study, we found that under different phases of the repeated low-dose CDDP treatment, Mettl3 expression was induced by two different mechanisms. In the presence of CDDP, the transcription factor Hif1-α was induced, resulting in an increase in Mettl3. When CDDP was removed, the previously increased Mettl3 caused an elevated lactate level, which formed a positive feedback loop by mutually reinforcing each other's expression via H3K18 lactylation. Functionally, we disclose that the knockout of Mettl3 in proximal tubules mitigates repeated low-dose CDDP-induced renal fibrosis both *in vitro* and *in vivo*. Mechanistically, Mettl3 stabilizes Pfkfb3 mRNA through N6-methyladenosine (m6A) modification and subsequently induces lactate production to upregulate the PD-L1 expression via H3K18 lactylation, thereby promoting both tumor growth and CDDP-induced renal damage. Intriguingly, we discovered that Levosimendan suppresses the methyltransferase activity of Mettl3 to lower the m6A level but has no impact on the abundance of the Mettl3-Mettl14 complex. PLGA-encapsulated Levosimendan not only alleviates repeated low-dose CDDP-induced renal fibrosis, but also significantly enhances the chemotherapeutic effects of cisplatin in several xenograft and syngeneic mouse tumor models by suppressing the Mettl3/Pfkfb3/lactate/ H3K18la/PD-L1 axis. Collectively, targeting Mettl3 might offer an effective therapeutic strategy during cisplatin-based chemotherapy-induced renal fibrosis, and PLGA-encapsulated Levosimendan is a potential intervention approach.

## Introduction

The kidney plays a pivotal role in the elimination of xenobiotics, and kidney injury is an inevitable consequence of drug treatment^1^. Cisplatin (CDDP), a DNA cross-linking agent with anti-tumor properties, induces the DNA damage response (DDR) and subsequent cell death^2^, ^3^. Unfortunately, cancer patients receiving multiple treatments with cisplatin developed chronic kidney disease, characterized by renal tubulointerstitial fibrosis^4^. To date, there is no effective treatment for CKD except for renal replacement therapy. Although two studies revealed that MDM2 and p53 were involved in CDDP-induced CKD^5^, ^6^, the underlying mechanism remains largely unknown. In addition, identifying a therapeutic target that can halt the progression of tubulointerstitial fibrosis and enhance the efficacy of CDDP is crucial for its clinical application.

Post-transcriptional RNA modifications regulate RNA splicing, transportation, and degradation^7^. The N6-methylation of adenosine (m6A), a prevalent RNA modification, influences various biological and pathological processes^8^. Methyltransferases form a complex that catalyzes m6A occurrence, with Methyltransferase 3 (Mettl3) being a crucial component involved in both malignancies and nephropathies^9^. In several cancers, Mettl3 acts as an oncogene by modulating m6A modifications of target genes, thereby controlling their expression or stability. For instance, Mettl3 promotes the tumorigenesis of breast cancer by increasing LATS1^10^. Other studies have also found that Mettl3 induces the proliferation of bladder, ovarian, and pancreatic cancer cells^11^-^14^. Mettl3 is also engaged in the pathogenesis of multiple nephropathies including AKI^15^, ^16^ and renal fibrosis^17^, ^18^. Based on the literature, we hypothesize that Mettl3 inhibition could reduce CDDP-induced renal fibrosis and improve CDDP efficacy.

In the current study, we found for the first time that the inhibition or proximal tubular deletion of Mettl3 attenuates low-dose CDDP-induced kidney damage and enhances the chemotherapeutic efficacy both *in vivo* and *in vitro*. Mechanistically, Mettl3 stabilizes Pfkfb3 mRNA, a key enzyme in glycolysis, via m6A modification. Accelerated glycolysis causes lactate accumulation, activating kidney fibroblasts and promoting tumor growth through PD-L1 upregulation. Furthermore, the excessive lactate induces the expression of Mettl3 through lactylation, which subsequently enhances the expression of Pfkfb3, thereby forming a positive feedback loop. Targeting Mettl3 might provide a novel therapeutic strategy to prevent renal fibrosis during cisplatin-based chemotherapy, and PLGA-encapsulated Levosimendan represents a promising therapeutic approach.

## Results

### Repeated low-dose cisplatin treatment led to the expression of Mettl3 and renal fibrosis both *in vitro* and *in vivo*

**Figure 1:**
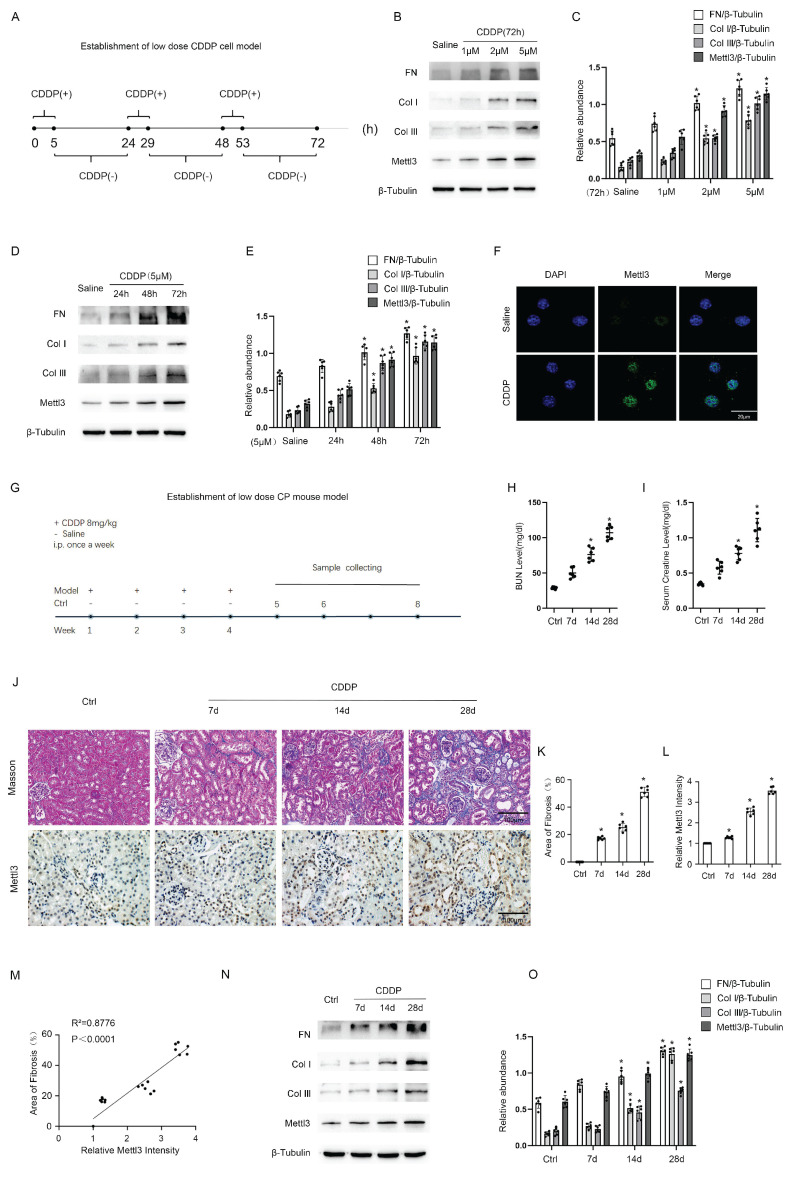
** The establishment of repeated low-dose CDDP-induced AKI-CKD models both *in vitro* and *in vivo*.** A. Schematic diagram depicting the establishment of the *in vitro* model. Briefly, for each day within 1-3days, BUMPT cells are cultured in the medium with a low dose (1,2,5μM) of CDDP for 5 hours and then in the normal medium for 19 hours. B&C. Western blot analysis for Mettl3 and fibrotic markers FN, Col I & Col III in BUMPT cells treated with saline or 1,2,5μM CDDP for 3 days, with β-Tubulin as the internal reference. D&E. Western blot analysis for Mettl3 and fibrotic markers FN, Col I & Col III in BUMPT cells treated with saline or 5μM CDDP for 1,2,3 days, with β-Tubulin as the internal reference. F. Immunofluorescence staining of Mettl3. (scale bar: 20 μm). G. Schematic diagram depicting the establishment of the *in vivo* model. Briefly, grouped mice are either intraperitoneally injected with 8mg/kg CDDP or the same volume of saline once a week for 4 weeks, and then sacrificed 7,14 or 28 days later for sample collection. H&I, Measurements of renal function indicators BUN and serum creatinine. J-L. Masson's trichrome staining presenting the area of kidney fibrosis (scale bar: 100 μm) and immunohistochemical staining presenting the expression level of Mettl3 (scale bar: 100 μm). M. Correlation analysis of Mettl3 positive percentage and fibrotic area. N&O. Western blot analysis for Mettl3 and fibrotic markers FN, Col I & Col III in mice kidney samples collected 7, 14 or 21 days after 4 weeks of CDDP(8mg/kg) treatment, with β-Tubulin as the internal reference. Data are expressed as mean ± SD (n = 6). *P < 0.05 versus saline or control.

To simulate renal tubular injury in cancer patients undergoing chemotherapy, we established *in vitro* models by exposing BUMPT cells to low-dose CDDP for 5 hours daily over a consecutive 72-hour period, as detailed in Fig. 1A. Immunoblot analysis demonstrated that CDDP induced the expression of FN, collagen I, collagen III, and Mettl3 in a dose-dependent manner at concentrations of 1 μM, 2 μM, and 5 μM (Fig. 1B & C). Furthermore, immunoblot results demonstrated that 5 μM CDDP progressively enhanced the expression of FN, collagen I, collagen III, and Mettl3 over an extended time course of 24, 48, and 72 hours (Fig. 1D & E). Based on these findings, we selected 5 μM CDDP as the intervention dose and 72 hours as the intervention duration. At 72 hours, 5μm CDDP significantly induced the expression of Mettl3, which was primarily localized in the nucleus of renal tubular cells, as indicated by immunofluorescence staining (Fig. 1F). We further developed *in vivo* models by peritoneally injecting CDDP into C57BL/6 mice at 8mg/kg once a week for 4 consecutive weeks, and then collected kidney samples on the subsequent 7th, 14th, and 28th days, as shown in Fig.1G. Elevated BUN and Cr levels indicated a decline in renal function after repeat low-dose CDDP treatment (Fig. 1 H&I), while Masson staining demonstrated increased interstitial fibrosis (Fig. 1J&K). The immunohistochemistry staining indicated that repeat low-dose CDDP treatment triggered the expression of Mettl3 in mice kidneys in a time-dependent fashion and confirmed the localization of Mettl3 in the nucleus of renal tubular cells (Fig. 1J&L). Correlation analysis disclosed a positive association between Mettl3 expression and fibrotic markers (Fig.1 M). Immunoblot analysis verified that low-dose CDDP treatment triggered the expression of FN, collagen I, and collagen III, as well as Mettl3 in a time-dependent manner, in line with the *in vitro* observations (Fig. 1 N&O). Collectively, our data suggests that low-dose CDDP induces renal fibrosis and upregulates Mettl3 expression.

### Mettl3 mediates renal fibrosis triggered by repeated low-dose CDDP in BUMPT cells

**Figure 2:**
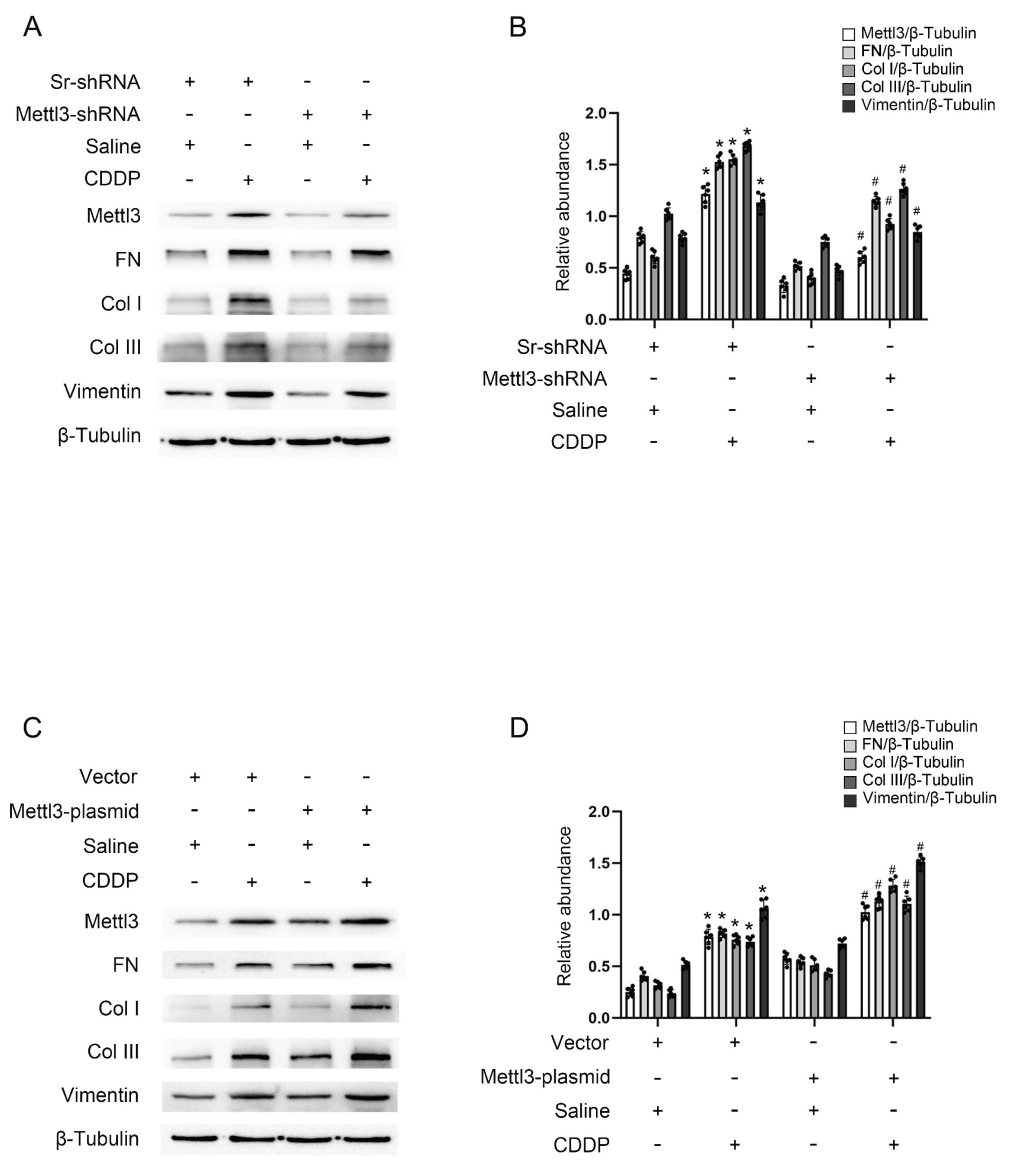
** Mettl3 mediates repeated low-dose CDDP-induced tubular cell fibrosis.** For suppression of Mettl3 expression, stable cell line of Mettl3 knockdown by lentivirus delivered shRNA was constructed. A&B. Western blot analysis for Mettl3 and fibrotic markers FN, Vimentin, Col I & Col III in Sr-shRNA transfected cell line and Mettl3-shRNA transfected cell line treated with saline or 5μM CDDP for 3 days. To enhance Mettl3 expression, the Mettl3 plasmid was transfected into BUMPT cells before CDDP treatment. C&D. Western blot analysis for Mettl3 and fibrotic markers FN, Vimentin, Col I & Col III in BUMPT cells treated with saline or 5μM CDDP for 3 days. Data are expressed as mean ± SD (n = 6). *P < 0.05 versus saline. #0.05 versus Sr-shRNA/CDDP or Vector/CDDP.

To investigate the role of Mettl3 in renal fibrosis induced by repeated low-dose CDDP, either a stable cell line with Mettl3-shRNA or a Mettl3 plasmid transfection was used to modulate Mettl3 expression. Fluorescence microscopy demonstrated significant transfection efficiency of both GFP-vector and GFP-Mettl3-shRNA (Fig. S1A). Immunoblot analysis revealed that repeated low-dose CDDP (5μM) increased the levels of Mettl3, FN, Vimentin, collagen I, and collagen III. This increase was attenuated by Mettl3 knockdown but enhanced by Mettl3 overexpression (Fig. 2A-D). The data indicated that Mettl3 acts as a promoter of fibrosis under repeated low-dose CDDP conditions.

### Mettl3 enhances Pfkfb3 mRNA stability by modulating m6A modification

**Figure 3:**
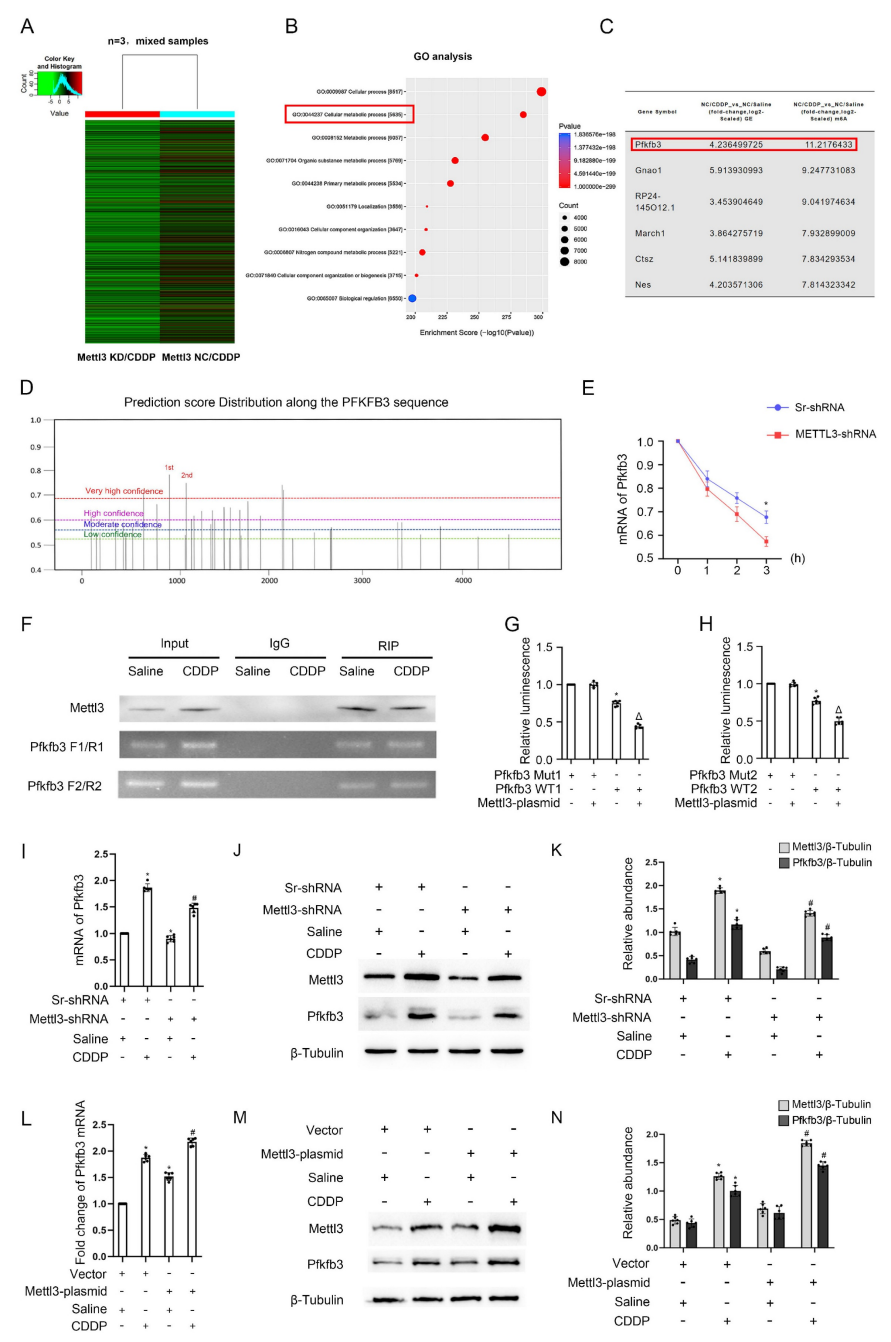
** Mettl3 regulates the expression of Pfkfb3 through m6A modification.** 3 groups of differently treated cells (NC / Saline, NC / CDDP, and Mettl3 KD/ CDDP) were used for RIP-seq analysis. A. The heatmap depicting genes' expression level (n=3, mixed samples). B. Go analysis. C. Differentially expressed mRNAs (fold change>3). D. Prediction of m6A binding sites along the Pfkfb3 sequence. 5μg/mL E. Actinomycin-D was introduced into the Sr/Mettl3-shRNA stable cell lines, and then the Pfkfb3 mRNA level at the indicated time points were detected through RT-qPCR analysis. *P < 0.05 versus Sr-shRNA. F. RIP-PCR of the two predicted binding sites with the highest score. G&H. Luciferase activity detection following co-transfection. *P < 0.05 versus Pfkfb3 Mut group. ΔP<0.05 versus other groups. I, J&K. RT-qPCR and Western blot analysis for Mettl3 and Pfkfb3 in Sr-shRNA or Mettl3-shRNA cell lines with or without CDDP treatment. L, M&N. RT-qPCR and Western blot analysis for Mettl3 and Pfkfb3 in vector or Mettl3 plasmid transfected BUMPT cells with or without CDDP treatment. Data are expressed as mean ± SD (n = 6). *P < 0.05 versus saline. # P <0.05 versus Sr-shRNA/CDDP or Vector/CDDP.

As is known, Mettl3 typically mediates the m6A modification of mRNAs, thereby increasing the stability of downstream genes^19^-^21^. To investigate the role of Mettl3 in fibrosis induced by repeated low-dose CDDP, we subjected three cell groups (NC/Saline, NC/CDDP, and Mettl3 KD/CDDP) to MeRIP/m6A sequencing, followed by bioinformatic analysis. We applied a filter of fold change ≥ 3 to identify genes that were upregulated in the NC/CDDP group compared to the NC/Saline group (Table S1) and downregulated in the Mettl3 KD/CDDP group compared to the NC/CDDP group (Table S2). Figure 3A presents a heatmap of differentially expressed genes in the Mettl3 KD/CDDP group compared to the NC/CDDP group. GO analysis showed that these genes are closely linked to cellular metabolic processes (Fig. 3B). From these results, we identified six genes that were upregulated by CDDP and downregulated by Mettl3 knockdown, with Pfkfb3 exhibiting the relatively higher fold change and a more specific role in metabolic processes (Fig. 3C). Additionally, recent research has revealed that Pfkfb3 interacts with Cdk4 to mediate cisplatin-induced tubular cell death^22^. The prediction of m6A-modification sites (http://www.dcuilab.cn/sramp) identified five reliable m6A sites on the Pfkfb3 mRNA sequences (Fig. 3D). To validate the effect of Mettl3 on Pfkfb3 stability, we treated stable Scramble-shRNA and Mettl3-shRNA cell lines with 5 μg/mL actinomycin-D. The cells were then collected for analysis at various time points. The data indicated that the inhibition of Mettl3 decreased the stability of Pfkfb3 at 2 and 3 hours (Fig. 3E). Subsequently, the top 2 sites were selected for RIP, confirming the interaction between Mettl3 and Pfkfb3 (Fig. 3F). The dual luciferase reporter assay demonstrated that Mettl3 significantly inhibited the luciferase activity of the wild-type Pfkfb3 plasmids, but not the plasmids with mutated m6A sites (Fig. 3 G&H), further confirming the interaction between Mettl3 and Pfkfb3 mRNA at the binding site. Additionally, RT-qPCR and immunoblot analyses showed that Mettl3 knockdown reduced the mRNA and protein levels of Pfkfb3, while Mettl3 overexpression increased these levels under saline conditions and with repeated low-dose CDDP (5 μM) (Fig. 3 I-N). In conclusion, the data indicates that Mettl3 enhances the stability of Pfkfb3 mRNA by promoting m6A modification.

### Pfkfb3 mediates repeated low-dose CDDP-induced renal fibrosis in BUMPT cells

**Figure 4:**
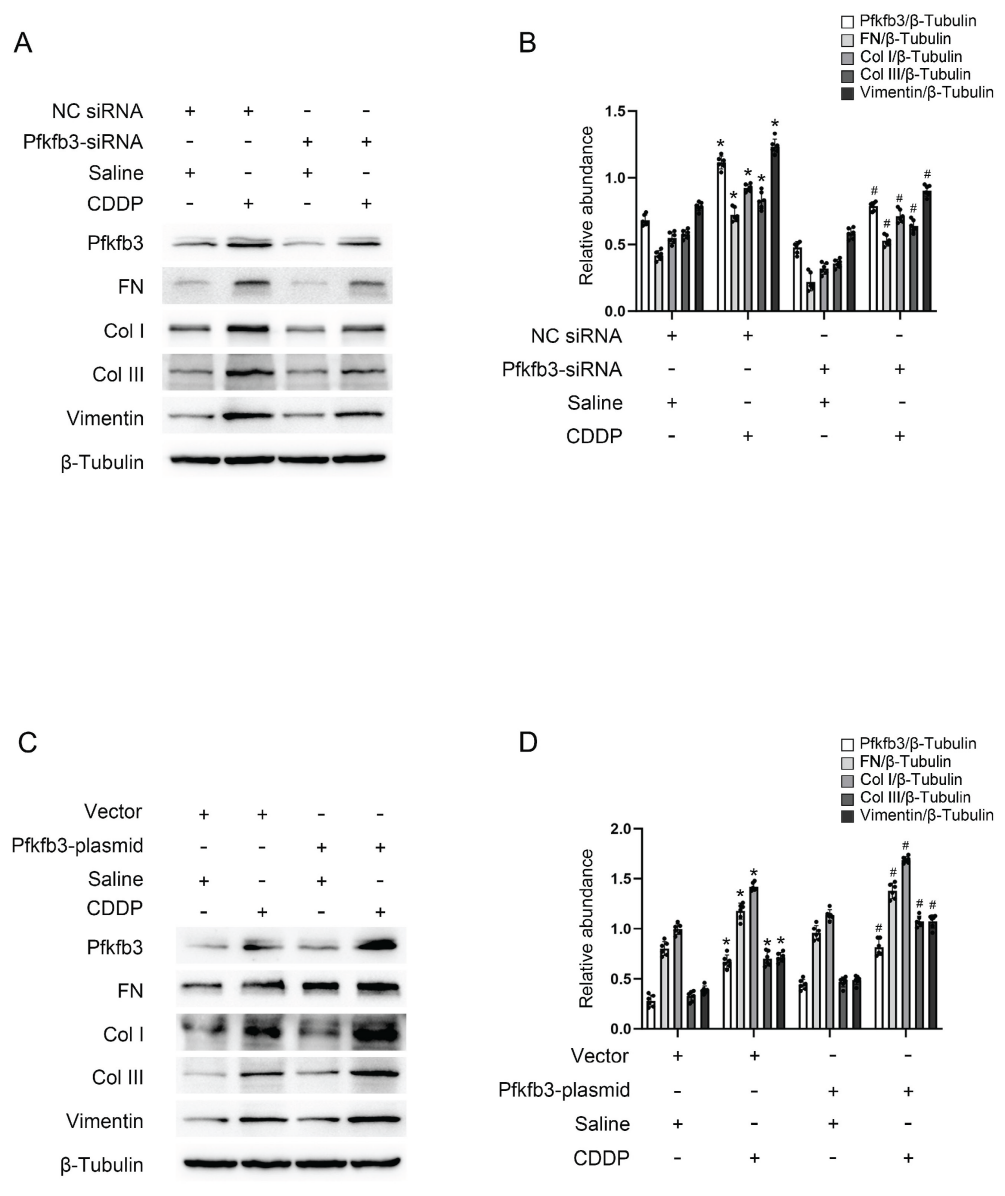
** Pfkfb3 mediates repeated low-dose CDDP-induced tubular cell fibrosis.** Pfkfb3 siRNA or plasmid was transfected into BUMPT cells before CDDP treatment. A&B. Western blot analysis for Pfkfb3 and fibrotic markers FN, Vimentin, Col I & Col III in nonsense control (NC) siRNA or Pfkfb3 siRNA transfected BUMPT cells treated with saline or 5μM CDDP for 3 days. C&D. Western blot analysis for Pfkfb3 and fibrotic markers FN, Vimentin, Col I & Col III in vector or Pfkfb3 plasmid transfected BUMPT cells treated with saline or 5μM CDDP for 3 days. Data are expressed as mean ± SD (n = 6). *P < 0.05 versus NC/saline or vector/saline. #P < 0.05 versus NC/CDDP or Vector/CDDP.

Pfkfb3 siRNA and plasmid were transfected into BUMPT cells to modulate its expression for the purpose of validating its function. As anticipated, immunoblot analysis revealed that the inhibition of Pfkfb3 decreased fibrotic markers such as FN, Vimentin, collagen I, and collagen III in BUMPT cells treated with repeated low-dose CDDP (Fig. 4A&B), while the over-expression of Pfkfb3 increased these markers (Fig. 4C&D). Pfkfb3 plays a detrimental role in the progression of kidney fibrosis.

### Pfkfb3-mediated lactate production in BUMPT cells under repeated low- dose CDDP treatment activates mouse kidney fibroblasts

**Figure 5:**
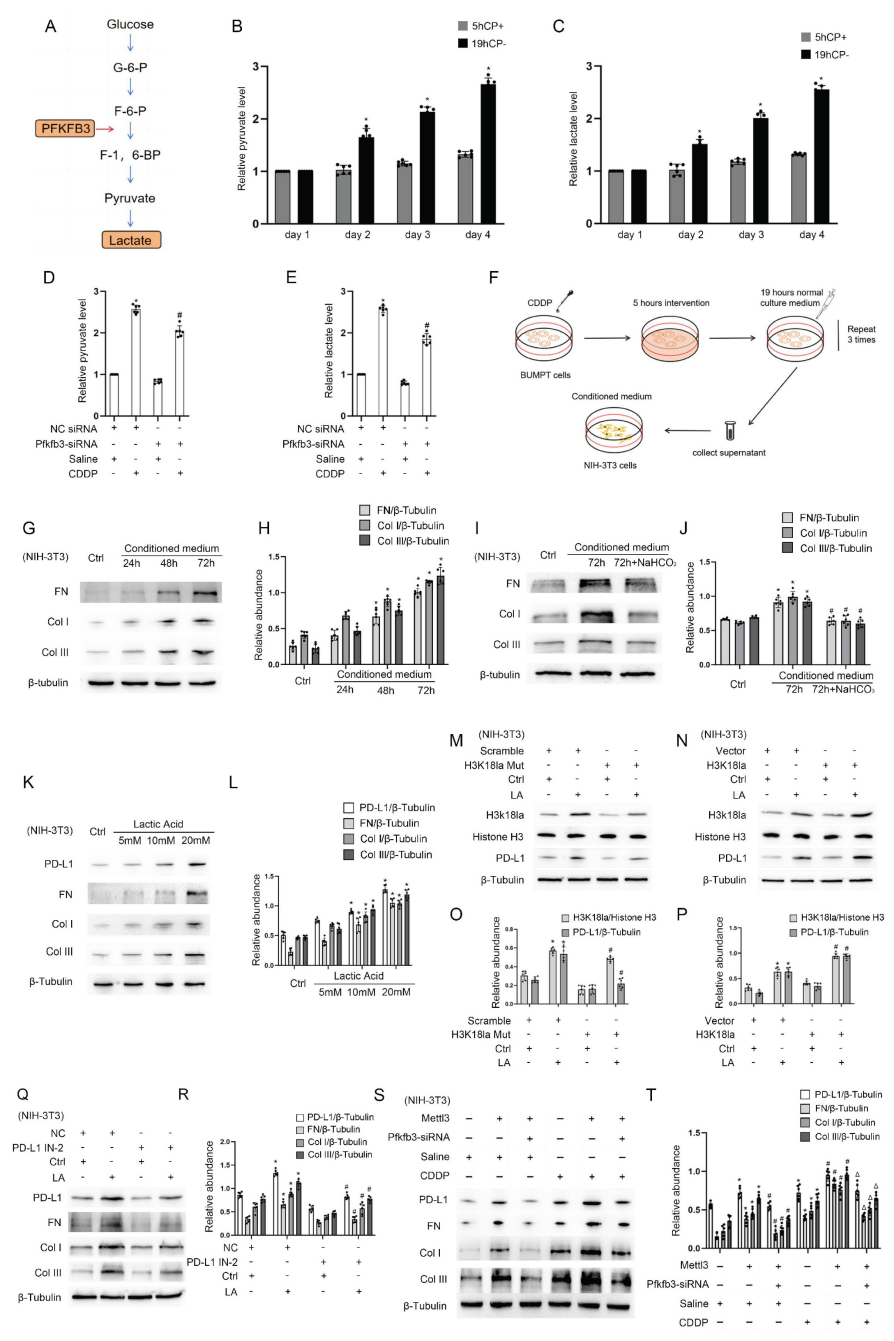
** Pfkfb3 regulates renal cell fibrosis via lactate/H3K18la/PD-L1 axis.** A. Schematic diagram depicting the role of Pfkfb3 in the lactate production process during glycolysis. B. Relative medium pyruvate level of BUMPT cells in different phases of the CDDP treatment. C. Relative medium lactate level of BUMPT cells in different phases of the CDDP treatment. *P < 0.05 versus day 1. D. Relative medium pyruvate level of BUMPT cells transfected with NC/Pfkfb3 siRNA plus CDDP treatment. E. Relative medium lactate level of BUMPT cells transfected with NC/Pfkfb3 siRNA plus CDDP treatment. F. Schematic diagram depicting the culture of NIH/3T3 cells using CDDP-treated BUMPT cell medium. G&H. Western blot analysis for fibrotic markers FN, Col I & Col III in NIH/3T3 cells treated with conditioned medium for 24, 48 and 72 hours. I&J. Western blot analysis for fibrotic markers FN, Col I & Col III in NIH/3T3 cells treated with conditioned medium or conditioned medium/NaHCO_3_ for 72 hours. K&L. Western blot analysis for PD-L1 and fibrotic markers FN, Col I & Col III in NIH/3T3 cells treated with lactate (5, 10, and 20 mM) for 24hours. M&O. Western blot analysis for Histone H3, H3k18la and PD-L1 in NIH/3T3 cells under lactate treatment after transfected with scramble/H3K18la mutant. N&P. Western blot analysis for Histone H3, H3k18la and PD-L1 in NIH/3T3 cells under lactate treatment after transfected with vector/H3K18la plasmid. Q&R. Western blot analysis for PD-L1 and fibrotic markers FN, Vimentin, Col I & Col III in NIH/3T3 cells under lactate treatment after PD-L1 inhibition. S&T. Western blot analysis for PD-L1 and fibrotic markers FN, Vimentin, Col I & Col III in NIH/3T3 cells under lactate treatment after co-transfection of Mettl3 plasmid and Pfkfb3 siRNA. Data are expressed as mean ± SD (n = 6). *P < 0.05 versus NC/saline. #0.05 versus plasmid/lactate. ΔP<0.05 versus Mettl3 plasmid/lactate group.

We then try to further understand how Pfkfb3 participates in the process of renal fibrosis. Pfkfb3, a key enzyme in the glycolysis process^23^, ^24^, regulates renal fibrosis and the production of pyruvate and lactate (Fig.5A-C). We for the first time reported that repeated low-dose CDDP (5μM) causes BUMPT cells to secrete more pyruvate and lactic acid in a time-dependent way (24, 48, and 72 hours) (Fig. 5B&C). Furthermore, we found that this upregulated secretion of pyruvate and lactate was suppressed by the knockdown of Pfkfb3 (Fig. 5D&E). The data show that Pfkfb3, downstream gene of Mettl3, promotes the progression of renal fibrosis as well as production of production of pyruvate and lactate during repeated low-dose CDDP. We collected the supernatant of BUMPT cells that had undergone repeated low-dose CDDP treatment and used it as a conditioned medium to culture NIH-3T3 cells, as depicted in Fig. 5F. The results indicated that this conditioned medium promoted the expression of FN, collagen I, and collagen III in NIH-3T3 cells in a time-dependent manner (Fig. 5G&H). To determine whether lactate in the conditioned medium was responsible for the observed effects, we added NaHCO_3_ to the conditioned medium to neutralize lactic acid. This treatment led to a reduction in the levels of fibrotic markers, as demonstrated in Fig. 5I&J. A recent study suggested lactate boosts PD-L1 expression in lung cancer^25^, while another indicated that PD-L1 inhibits vimentin degradation to exacerbate pulmonary fibrosis^26^. Our data further confirmed that lactate increases the expression of FN, collagen I, collagen III, and PD-L1 in fibroblasts in a dose-dependent manner (5, 10, and 20 mM) (Fig. 5K&L). Lactate induces histone lactylation, which regulates gene expression^27^-^29^. To investigate the effect on PD-L1 expression, we altered the histone lactylation levels in NIH-3T3 cells by either mutating H3K18la or using a plasmid. The results showed a positive linear relationship between PD-L1 expression and the H3K18la level (Fig. 5M-P). Notably, inhibiting PD-L1 reduced the lactate-induced expression of FN, collagen I, and collagen III, indicating that PD-L1 mediates lactate's profibrotic effects in fibroblasts (Fig. 5O&R). Lastly, immunoblot analysis showed that supernatant from BUMPT cells transfected with Mettl3 enhanced the expression of FN, collagen I, and collagen III in NIH-3T3 cells. This effect was significantly reduced when BUMPT cells were transfected with Pfkfb3 siRNA, both in the presence and absence of lactate (Fig.5S&T). Collectively, these results indicate that CDDP-induced Mettl3 expression raises Pfkfb3 levels, thereby promoting glycolysis and lactate secretion in tubular epithelial cells. Subsequently, excessive lactate induces histone lactylation in renal fibroblasts, which increases PD-L1 levels and promotes fibrosis. Thus, the Mettl3/Pfkfb3/lactate/ H3K18la/PD-L1 axis regulates renal fibrosis induced by low-dose cisplatin.

### Mettl3 expression with or without CDDP treatment is respectively induced by Hif1-α or lactate

**Figure 6:**
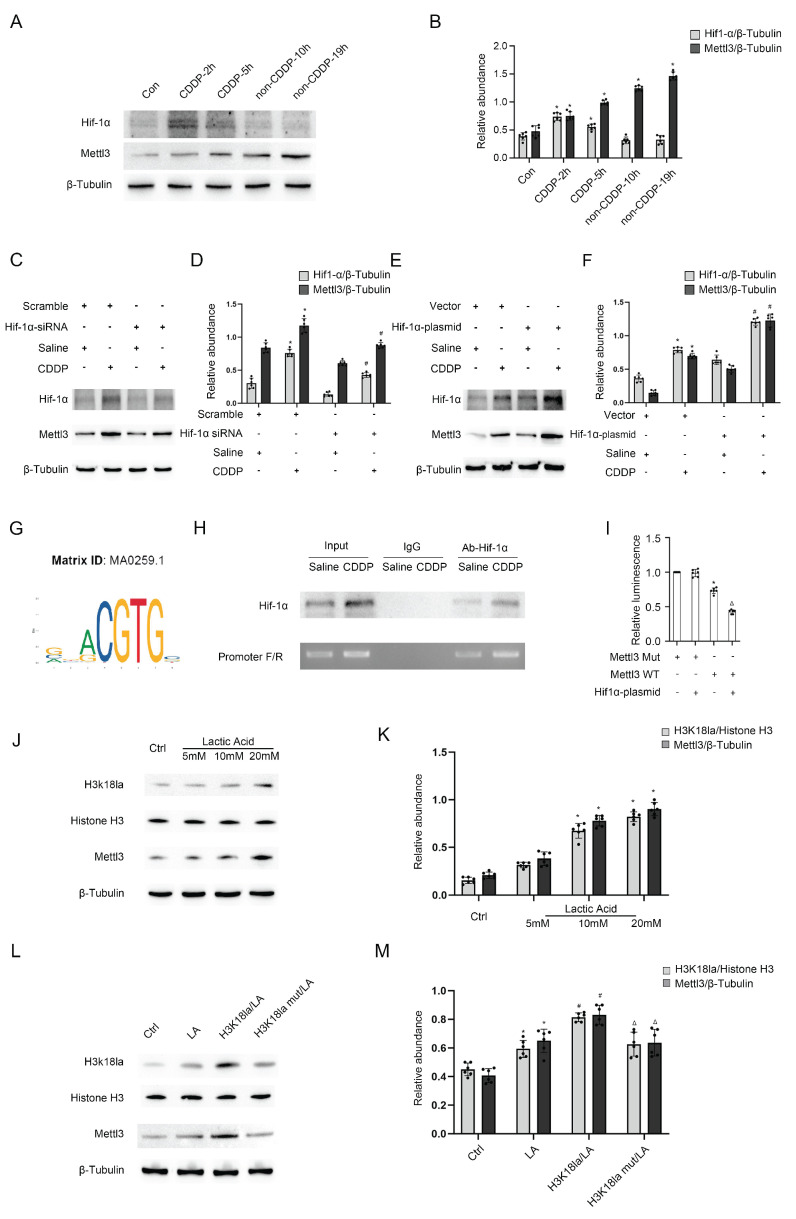
** Both Hif1-α and H3K18la lactylation mediates Mettl3 expression during CDDP treatment.** A&B. Western blot analysis for Hif1-α in different phases of the CDDP treatment. C&D. Western blot analysis for Hif1-α and Mettl3 in BUMPT cells treated with saline or CDDP (5μM, 2h) after NC/Hif1-α siRNA transfection. E&F. Western blot analysis for Hif1-α and Mettl3 in BUMPT cells treated with saline or CDDP (5μM, 2h) after vector/Hif1-α plasmid transfection. G. Illustration of predicted binding site of Hif1-α and Mettl3 promoter region. H. ChIP-PCR analysis of the binding site using Hif1-α antibody. I. Luciferase activity detection following co-transfection. *P < 0.05 versus Mettl3 Mut group. ΔP < 0.05 versus other groups. J&K. Western blot analysis for Histone 3, H3K18la and Mettl3 in BUMPT cells treated with lactate (5, 10, and 20 mM) for 24hours. L&M. Western blot analysis for Histone H3, H3K18la and Mettl3 in BUMPT cells treated with 20mM lactate after H3K18la mutant or plasmid transfection. Data are expressed as mean ± SD (n = 6). *P < 0.05 versus NC/saline. #0.05 versus plasmid/lactate.

Former results demonstrated that both CDDP treatment (5h/24h) and the absence of CDDP treatment (19h/24h) resulted in the expression of Mettl3. We were interested in the upstream mechanism. The well-known transcription factor Hif1-α was induced in BUMPT cells during the 5-hour period with CDDP treatment, but not during the subsequent 19-hour period without CDDP treatment (Fig. 6A&B). We modified Hif1-α using Hif1-α siRNA or plasmid, observing changes in Mettl3 expression (Fig. 6C-F), which suggests that Hif1-α regulates Mettl3 expression. The binding site of Hif1-α and the Mettl3 promoter region was predicted using JASPAR (https://jaspar.elixir.no/). ChIP PCR and DLR analysis jointly verified the interaction between Hif1-α and the Mettl3 promoter region (Fig. 6G-I). Considering the previous results, we hypothesize that lactate-induced lactylation contributed to Mettl3 expression in the 19 hours without the influence of Hif1-α. Immunoblot analysis confirmed our anticipation by showing an increased H3K18la and Mettl3 expression in tubular cells treated with lactate (Fig. 6J&K). Firstly, H3K18la and Mettl3 expression increased in tubular cells that underwent lactate treatment in a dose-dependent manner (Fig. 6J&K). Secondly, the alteration of H3K18la expression level affected Mettl3 expression under lactate treatment (Fig. 6L&M). Collectively, these results indicated that in the repeated low-dose CDDP treatment, Mettl3 expression is under two different regulatory mechanisms in distinct phases. Interestingly, Mettl3 and lactate form a positive feedback loop by enhancing each other's expression.

### Deletion of Mettl3 in proximal tubular cells alleviates CDDP-induced renal fibrosis by suppressing the Pfkfb3/lactate/ H3K18la/PD-L1 axis

**Figure 7:**
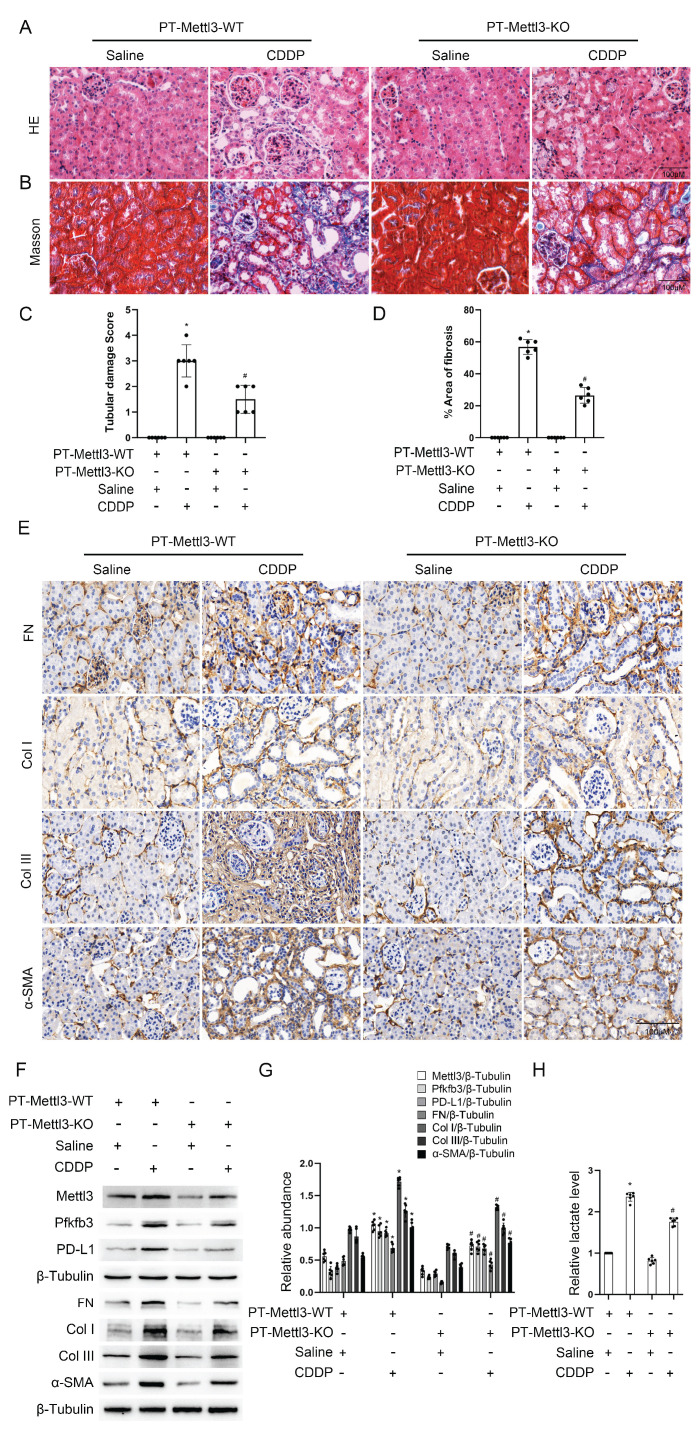
** Proximal tubular deletion of Mettl3 ameliorates CDDP-induced renal fibrosis.** Littermate PT-Mettl3-WT and PT-Mettl3-KO mice of the same age and weight were grouped for saline or CDDP intraperitoneal injection as previously described. A. H&E staining. B. Masson's trichrome staining. C. Tubular damage score. D. Area of fibrosis. E. IHC staining of fibrotic markers FN, Col I, Col III and α-SMA. F&G. Western blot analysis for Mettl3, Pfkfb3, PD-L1 and fibrotic markers FN, Col I, Col III and α-SMA in mice kidneys from different groups. H. Relative lactate level of the kidney. Data are expressed as mean ± SD (n = 6). *P < 0.05 versus PT-Mettl3-WT/saline. #0.05 versus PT-Mettl3-WT/CDDP.

To validate the *in vitro* findings, male littermate PT-Mettl3-wild-type (WT) and PT-Mettl3-KO mice of the same age were randomly assigned to control and model groups. These mice were then exposed to low-dose CDDP (8mg/kg) once a week for four consecutive weeks. H&E and Masson's trichrome staining showed that PT-Mettl3-KO mice had reduced renal tubular damage and fibrosis following low-dose CDDP treatment (Fig. 7A-D). Immunohistochemical staining results showed that PT-Mettl3-KO reduced the expression of FN, collagen I, collagen III, and α-SMA induced by low-dose CDDP (Fig. 7E). Immunoblot and lactate assay showed that PT-Mettl3-KO decreased the low-dose CDDP-induced expression of Mettl3, Pfkfb3, PD-L1 and fibrotic markers (Fig. 7F&G), and reduced lactate production (Fig. 7H). These data indicate that PT-Mettl3-KO mitigates renal fibrosis induced by low-dose CDDP.

### Levosimendan improves low-dose CDDP-induced renal fibrosis

**Figure 8:**
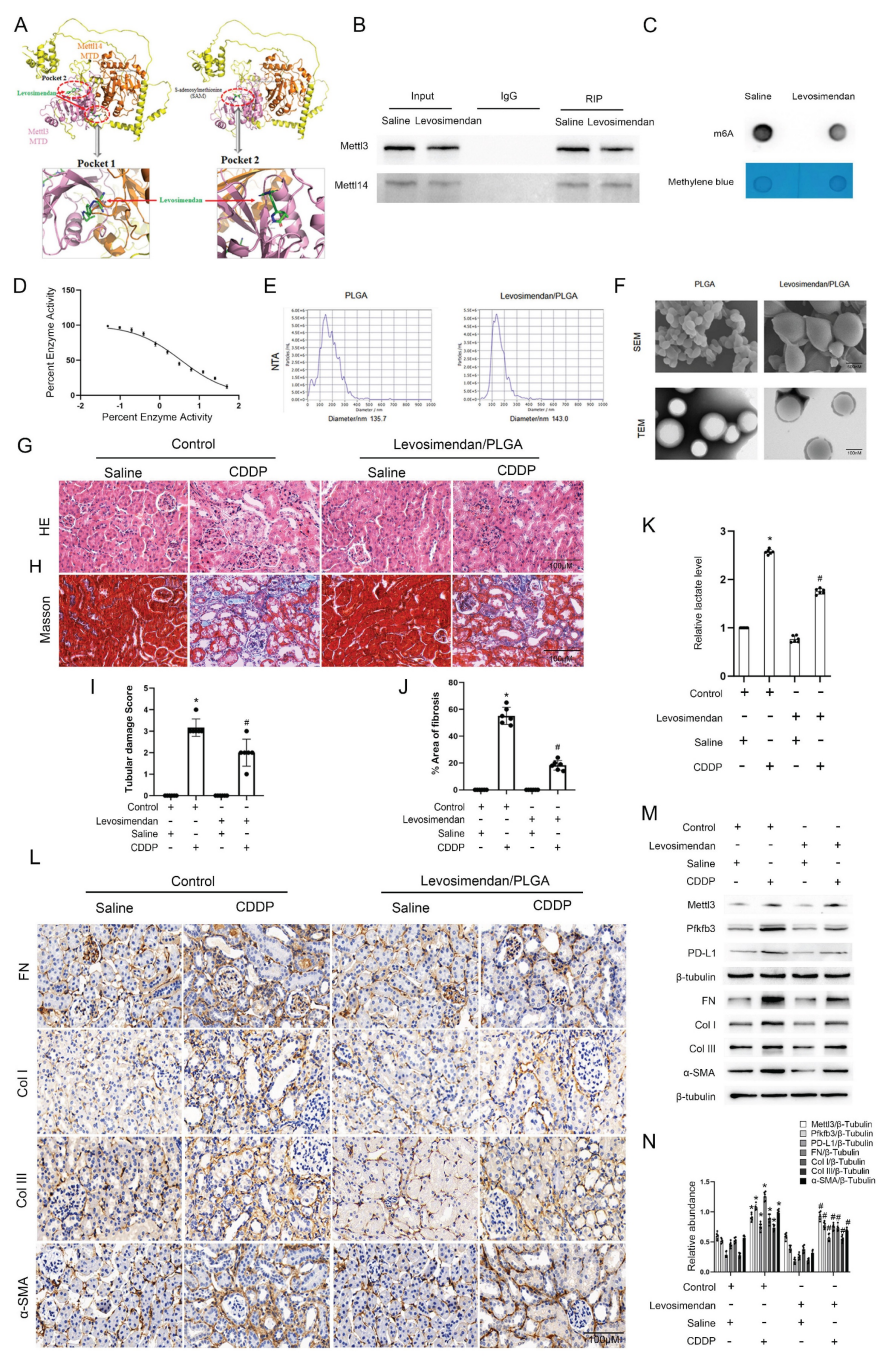
** PLGA encapsulated Levosimendan mitigates CDDP-induced renal fibrosis.** No load or Levosimendan loaded PLGA nanoparticles were injected into grouped (Saline/CDDP) littermate male C57BL/6 mice of the same age and weight. A. Structural prediction of the interaction between Mettl3-Mettl14 and Levosimendan. B. IP assay of Mettl3 and Mettl14 under Levosimendan interference. C. m6A dot blot of BUMPT cells under saline or Levosimendan treatment. D. Methytransferase activity assay. E. NTA measurement of no load PLGA and Levosimendan/PLGA. F. SEM and TEM observation of no load PLGA and Levosimendan/PLGA. G. H&E staining. H. Tubular damage score. I. Masson's trichrome staining. J. Area of fibrosis. K. IHC staining of fibrotic markers FN, Col I, Col III and α-SMA. F&G. L. Western blot analysis for Mettl3, Pfkfb3, PD-L1 and fibrotic markers FN, Col I, Col III and α-SMA in mice kidneys from different groups. Data are expressed as mean ± SD (n = 6). *P < 0.05 versus PLGA/saline. #0.05 versus PLGA/CDDP.

Using small molecule compounds to inhibit target gene expression represents a promising approach for disease intervention. Levosimendan is a clinically approved drug mainly utilized for managing heart failure as a calcium sensitizer and inodialator. We predicted two possible pockets in the Mettl3-Mettl14 complex that could accommodate Levosimendan (Fig. 8A). The binding of Levosimendan to these sites has the potential to disrupt the methyltransferase activity of Mettl3. Dot blot analysis showed that a 2.5μM concentration of Levosimendan, incubated for 24 hours, reduced the m6A level in BUMPT cells (Fig. 8C). Co-IP analysis demonstrated that levosimendan does not interfere with the formation of the Mettl3-Mettl14 complex (Fig. 8B), while the methyltransferase activity assay indicated that it suppressed the enzyme activity of the Mettl3-Mettl14 complex in a dose-dependent manner (Fig. 8D). NTA showed the average diameter of PLGA microspheres as 135.7nm and that of Mettl3/PLGA as 143.0nm (Fig. 8E). We encapsulated Levosimendan with PLGA nano particles to boost its efficacy *in vivo*. Successfully prepared Levosimendan/PLGA microspheres were indicated by SEM and TEM (Fig. 8F). H&E and Masson staining results demonstrated that intravenous administration of Levosimendan/PLGA via the tail vein significantly alleviated cisplatin (CDDP)-induced renal damage and fibrosis (Fig. 8G-J). The lactate assay revealed that Levosimendan/PLGA reduced CDDP induced lactate increase (Fig. 8K). Furthermore, immunohistochemical staining and immunoblot collectively showed that Levosimendan/PLGA suppressed the expression of Mettl3, Pfkfb3, and PD-L1, in CDDP-treated mice kidneys (Fig. 8L-N). Levosimendan exhibited a renoprotective effect in low-dose CDDP-induced AKI-CKD by inhibiting the Mettl3/Pfkfb3/lactate/PD-L1 pathway.

### Levosimendan enhances CDDP efficacy in xenograft models of ovarian, bladder, and breast cancer

**Figure 9:**
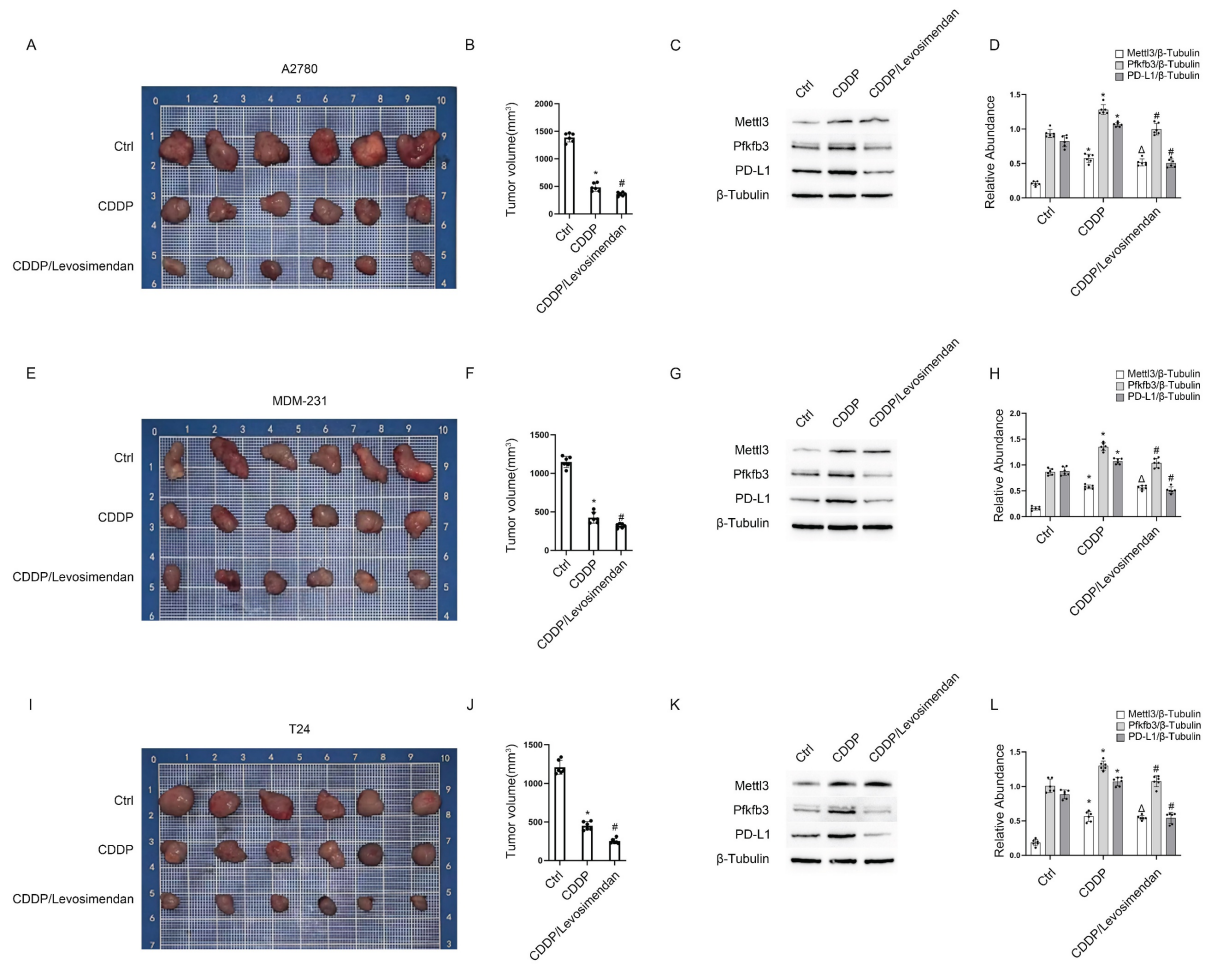
** PLGA encapsulated-Levosimendan enhances CDDP efficacy in xenograft models of ovarian, bladder, and breast cancer.** Nude mice were inoculated with A2780 ovarian cancer cells or MDM-231 breast cancer cells or T24 bladder cancer cells to build tumor xenografts. When the tumors reached approximately 200mm^3^, randomly group the nude mice into the PLGA group, the PLGA/CDDP group and the PLGA encapsulated- Levosimendan/CDDP group for subsequent weekly treatment. A, E&I. Representative images of the dissected tumors. B, F&J. Tumor volume measurements. CD, GH&KL. Western blot analysis of Mettl3, Pfkfb3 and PD-L1 in the dissected tumors. *P < 0.05 versus ctrl. #0.05 versus CDDP.

Three tumor cell lines—A2780 (ovarian cancer), MDA-MB-231 (breast cancer), and T24 (bladder cancer)—were injected into the flanks of nude mice to establish cancer xenografts. After 2 weeks, the tumors grew to approximately 200 mm^3^ in volume. Subsequently, each type of tumor in nude mice was divided into 3 groups for treatment: group I, saline; group II, CDDP; group III, CDDP plus Levosimendan/PLGA for 4 weeks. Then, blood, tumor, and kidney samples were collected to examine renal damage and the efficacy of chemotherapy. Data showed that CDDP significantly reduced tumor size in ovarian, bladder, and breast cancers, with further enhancement by CDDP plus Levosimendan/PLGA (Fig. 9). Immunoblot results showed increased Mettl3 expression in both group II and group III. However, PD-L1 expression rose in group II but fell in group III (Fig. 9C&F&I). This suggests that Levosimendan/PLGA disrupts Mettl3 methyltransferase activity, leading to a decrease in PD-L1 levels. Figure S2 showed less kidney damage in group III, as evidenced by lower BUN and blood creatinine levels and improved pathological staining, consistent with findings in C57BL/6 mice. In conclusion, Levosimendan enhances the efficacy of CDDP in treating tumors and reduces the reno-toxicity of CDDP.

## Discussion

Cisplatin (CDDP), an effective chemotherapeutic drug for solid tumors, is limited in clinical use due to side effects, particularly renal damage^30^. Limited research has reported the activation of certain signaling pathways during repeated CDDP exposure, primarily studied in cultured cells and animal models without tumors^5^, ^6^. The impact of reno-protective strategies on the chemotherapeutic efficacy of CDDP remains largely unknown. In the current study, we confirmed that Mettl3 plays a pivotal role in the development of CKD induced by low-dose CDDP. Additionally, in a nude mouse tumor-bearing model, data showed that Levosimendan, an inhibitor of Mettl3 methyltransferase activity, prevents CDDP-induced renal fibrosis and enhances CDDP chemotherapeutic efficacy. Overall, our findings elucidate a novel signaling mechanism underlying low-dose CDDP-induced CKD and identify a promising strategy for renoprotection in repeated low-dose CDDP-based cancer treatments.

Recent studies indicate Mettl3's role in renal disease progression. Our recent research showed that Mettl3 exacerbates I/R, sepsis, and vancomycin-induced AKI. This is achieved by enhancing the mmu-lncRNA 121686/hsa-lncRNA520657/miR-328-5p/HtrA3 axis^15^. Meng *et al.* also reported that Mettl3 exacerbates AKI and renal fibrosis, caused by unilateral ureteral obstruction (UUO) and ischemic reperfusion, by stabilizing TAB3 and EVL mRNA, respectively^16^, ^17^. Interestingly, Yu *et al.* found that Mettl3-induced m6A hypermethylation of the cGAS-STING pathway increases renal fibrosis^31^. Another study showed that Mettl3 promotes podocyte injury in diabetic nephropathy via TIMP2 mRNA m6A modification^18^. In the current study, we discovered that Mettl3 increases the stability of Pfkfb3 via m6A modification to induce the development of renal fibrosis (Fig. 3).

Recently, multiple studies have identified PFKFB3 as a key contributor to the progression of renal fibrosis. Chun *et al.* reported that elevated PFKFB3 expression contributes to hypoxia-induced kidney fibrosis^32^. Similarly, Mao *et al.* demonstrated that Pfkfb3 promotes glycolysis in proximal tubular cells during ischemia-reperfusion injury (IRI), leading to NF-κB family activation through lactylation^33^. Additionally, Pfkfb3-mediated glycolysis activates fibroblasts and promotes myeloid-associated inflammation, exacerbating renal fibrosis after unilateral ureter obstruction (UUO)^34^, ^35^. Another study demonstrated that Pfkfb3-mediated endothelial glycolysis contributes to diabetic kidney disease^36^. In our current study, we identified that Pfkfb3 regulates lactate production, which in turn induces PD-L1 expression and subsequently promotes fibrosis triggered by CDDP(Fig.4&5).

Mettl3 was induced by various factors, including STAT3, TBK1, H3K4me3, and EGR1^21^, ^37^-^39^. A recent study reported that lactate induces the expression of Mettl3 through H3K18 lactylation^40^. Here, we report a new finding and confirm that Mettl3 is initially induced by HIF-1α during CDDP treatment, and subsequently by H3K18la lactylation induced by lactate after CDDP removal. Therefore, we propose that HIF-1α acts as a switch, activating Mettl3 expression to produce lactate through Pfkfb3, which in turn promotes Mettl3 expression via lactylation, creating a positive feedback loop between lactate and Mettl3. Excess lactate in the tubular system activates 3T3 cells by upregulating pro-fibrotic PD-L1, contributing to CDDP-induced renal fibrosis (Fig 5), which was supported by the reference that PD-L1 mediates the organ fibrosis^26^, ^41^. Several inhibitors have been reported. For instance, STM2457 binds to the SAM - binding pocket of Mettl3, competitively inhibiting its activity, showing anti - leukemia effects in pre - clinical models and has entered phase I clinical trials^42^. RSM3, a stapled peptide inhibitor, not only inhibits Mettl3's catalytic activity but also induces its degradation via the proteasome pathway, showing anti-cancer effects in multiple tumor models^43^. Cpd-564 was identified as a METTL3 inhibitor exhibiting better protective effects against cisplatin-induced and ischemia/reperfusion-induced renal injury and inflammation compared with S-adenosyl-L-homocysteine, An effective inhibitor of SAM-dependent methyltransferase^16^, ^44^. However, most Mettl3 inhibitors are in pre-clinical research, and their clinical translation requires more studies on safety and efficacy. Repurposing old drugs offers advantages such as leveraging known safety profiles, established mechanisms of action, and clinical experience to significantly reduce development costs, shorten research cycles, and expand therapeutic applications. In the present study, we for the first time identified levosimendan, an approved anti- heart failure drug, as a new Mettl3 inhibitor, which supported by the dot blot assay and methyltransferase activity examination (Fig. 8A-D). Considering the short half-life of levosimendan *in vivo*, PLGA encapsulation was employed to overcome this limitation, as evidenced by the following: 1) PLGA-encapsulated Levosimendan mitigates the renal fibrosis caused by repeated low-dose CDDP treatment through the inhibition of the Mettl3/Pfkfb3/lactate/H3K18la/PD-L1 axis (Fig. 7&8). 2) In nude mice with tumors, Levosimendan not only down-regulated Pfkfb3 to reduce renal damage but also suppressed PD-L1, enhancing the efficacy of CDDP chemotherapy, which resulted in a reduction in tumor size (Fig.9&S2). Despite our findings, there are several key challenges in clinical translation of nanomedicines including unpredictable *in vivo* behavior, toxicity concerns, scalable production issues, poor targeting efficiency, lack of standardized regulatory criteria, and high costs limiting accessibility^45^, ^46^. Mettl3 is involved in the progression of several tumors and mainly exhibits oncogene property^47^-^49^, but showed anti-tumor function in endometrial cancer by targeting the AKT pathway^50^. Here, we provided evidence that Mettl3 promotes the progression of ovarian, breast and testicle cancers by enhancing the Pfkfb3/lactate/H3K18la/PD-L1 axis, and PLGA-encapsulated Levosimendan synergistically reduces tumor size in combination with CDDP. Tumors and fibrosis both exhibit metabolic abnormalities including energy metabolism reprogramming (enhanced glycolysis, suppressed mitochondrial oxidative phosphorylation, abnormal mitochondrial morphology), elevated glutamine catabolism, increased fatty acid synthesis and lactate accumulation^51^-^53^. They also exhibit distinct features and mechanisms: tumors are driven by oncogenes, and the goal is unrestricted proliferation and metastasis, while fibrosis is driven by chronic inflammation (TGF-β, IL-1β) and tissue injury, mostly leading to excessive ECM deposition and tissue repair^54^-^57^. PD-L1, a trans-membrane protein, suppresses the immune response when interacting with PD-1. Accumulating evidence indicates that the PD-1/PD-L1 axis is involved in various malignancies by facilitating the immune escape of cancer cells^58^-^60^. Therefore, the Mettl3/Pfkfb3/lactate/H3K18la/PD-L1 pathway was a common mechanism underlying the development of both renal fibrosis and tumor growth during repeated low-dose CDDP-based cancer therapy.

In summary, our study highlights Mettl3's role in CDDP-induced renal fibrosis, explains the underlying molecular mechanisms, and provides evidence that Mettl3 methyltransferase inhibition can both alleviate low-dose CDDP-induced renal fibrosis and enhance CDDP's tumor-killing efficiency. Mettl3 is a promising target for managing renal fibrosis during cisplatin therapy.

## Materials and Methods

### Antibodies & reagents

FN (Cat No. 15613-1-AP), Anti-Collagen I (Cat No. 14695-1-AP), Collagen III (Cat No. 22734-1-AP), Fibronectin (Cat No. 15613-1-AP), Vimentin (Cat No. 60330-1-Ig) and β-Tubulin (Cat No.10094-1-AP) antibodies were purchased from Proteintech Group, Inc (Rosemont, IL, USA). Mettl3 (#86132) antibody was purchased from Cell Signaling Technology, Inc (Danvers, MA, USA). PD-L1 (ab205921) and Histone H3(ab1791) were obtained from Abcam (Cambridge Science Park, Cambridge, UK). H3K18lac (PA5-116896) was bought from Invitrogen (Carlsbad, CA, USA). Mettl3 siRNA and Pfkfb3 siRNA were obtained from Ruibo (Guangzhou, China). Plasmids were constructed by Tsingke (Beijing, China). PD-L1-IN-2 (HY-149830) were bought from MCE China (Shanghai, China). Cisplatin (CAS number: 15663-27-1) was purchased from Sigma-Aldrich (Darmstadt, Germany).

### Cell culture and treatment

BUMPT (Boston university mouse proximal tubular) cells were obtained from Dr. Wilfred Lieberthal (Boston University School of Medicine), and then cultured in DMEM medium (Gibco, 11965092) complemented with 10% FBS (Gibco,10100147) and 1% Penicillin-Streptomycin (Gibco, 15140163). For repeated low-dose CDDP model, BUMPT cells were treated with CDDP for 5 hours then without CDDP for19hours for 3 times in a 72-hour period. In order to change the expression of certain genes, siRNA (5ng)/plasmid (2μg) were transfected using lipofectamine 2000 (Life Technologies, USA) at least 24 hours prior to following treatment.

The rationale behind our choice of BUMPT cells lies in their strong compatibility with animal models, low culture cost, high stability, and mature gene editing technology, which enables the rapid construction of stable knockout cell lines—all of which fully meet the requirements of this integrated *in vivo*-*in vitro* research. Human-derived cells such as HK-2, while having gene expression profiles and metabolic characteristics closer to the real human physiological environment and theoretically avoiding potential interference from species differences, may exhibit issues such as genetic mutations or phenotypic drift during *in vitro* culture. Additionally, when extrapolating *in vitro* results from human-derived cells to animal models, it is necessary to additionally validate gene expression differences between species, which undoubtedly increases the complexity and workload of the research.

In contrast, human-derived cells such as HK-2, while having gene expression profiles and metabolic characteristics closer to the real human physiological environment and theoretically avoiding potential interference from species differences, may exhibit issues such as genetic mutations or phenotypic drift during *in vitro* culture. Additionally, when extrapolating *in vitro* results from human-derived cells to animal models, it is necessary to additionally validate gene expression differences between species, which undoubtedly increases the complexity and workload of the research.

Stable Mettl3-KD cell line was constructed as following steps: First, the expression vector for the Mettl3 incorporating a promoter connecting selectable marker genes and multiple cloning sites was designed and synthesized by Tsingke (Beijing, China). This vector was then introduced into host cells via liposome-mediated transfection. Following transfection, cell clones that successfully integrated the foreign gene were selected under selective pressure through the addition of antibiotics. Finally, the expression of the Mettl3 was validated using PCR, Western blot analysis, and fluorescence microscopy.

### Animal model

C57BL/6J male mice (RRID: MGI:5657312) were obtained from Hunan SJA Laboratory Animal Co., Ltd., and housed on a 12-hour light/dark circle with free access to food and water. Repeated low-dose CDDP model was established by CDDP peritoneal injection once a week for 4 times, and biology samples were collected 1,2,4 weeks after the 4^th^ injection. The C57BL/6 mice was injected with or without Levosimendan/PLGA (20μg/kg equivalent of the drug) twice a week through tail vein at 1h after CDDP treatment, with PLGA as a control.

PT-Mettl3-KO mice were obtained as previously described^15^. In short, female heterozygous offspring (Mettl3 f/+XcreX) of male flox-flox mice harbouring Mettl3(Mettl3f/f XY) alleles and phosphoenolpyruvate carboxy kinase-cAMP response element (PEPCK-Cre) transgenic mice (Mettl3+/+ XcreXcre) were paired with Mettl3 f/fXcreY males to produce littermate PT-Mettl3-WT (Mettl3 +/+XcreY) and PT-METTL3-KO (METTL3 f/fXcreY) mice.

Different tumor cell lines were cultured and harvested for xenograft inoculation. Tumor cells quenched and re-suspended in saline were injected in either flank of male litter mate nude mice aged 6-7 weeks with similar weight.

### Immunofluorescence

Incubate the treated cells with Mettl3 (1:200) overnight at 4°C and then with the secondary fluorescent antibody for 1 h at 37°C in the dark. 3-5 minutes before observing the cells under fluorescent microscopy, add the nuclear marker DAPI.

### Real-time qPCR

The expression level of mRNAs was detected by RT-qPCR analysis. In brief, total RNA was extracted from bio-samples using RNAiso Plus (TaKaRa, T9108), and then reverse-transcribed into cDNA using the Evo M-MLV kit (AG, AG11705). The cDNA obtain was then amplified through PCR using SYBR Green Premix Pro Taq HS qPCR Kit (AG11701) and detected by Lightcycler (Roche) for relative quantification analysis using ΔΔCt values. GAPDH and U6 primers were described in a previous report^61^, ^62^.

### H&E Staining, Masson's trichrome staining and IHC

Kidney tissue was harvested and fixed for various pathological staining as we previously described^62^, ^63^. The tissue damage was estimated by hematoxylin and eosin staining. The degree of renal interstitial fibrosis was evaluated by Masson's trichrome staining. Expression levels of fibrotic markers were detected by immunohistochemistry with anti-FN (1:50 dilution), Col I (1:100 dilution), Col III (1:100 dilution), and α-SMA (1:50 dilution) antibodies. Quantification methods were described in our previous work^64^.

### Immunoblotting

Immunoblotting was conducted as mentioned in our previous work^65^, ^66^. Cells or tissues were lysed for protein collection. Treated protein underwent electrophoresis in SDS-PAGE gels and was transferred to PVDF membranes. After blocking with 5% skim milk, these membranes were incubated with primary antibodies (Mettl3 1:2000, FN 1:1000, Col I 1:1000, Col III 1:1000, H3K18lac 1:1500) at 4°C overnight. Then the membranes were incubated in corresponding secondary antibodies for 1 hour at room temperature. Specific protein bands were observed in a gel imager (Tanon, 4800) using ECL reagent. Between each step, the membranes needed adequate PBST washing. ImageJ software was used for quantification.

### Metabolite assay

Pyruvate and lactate level were detected using a Pyruvic Acid (PA) Content Detection Kit (BC2205, Solarbio) and Lactic Acid assay kit (A019-2-1, Nanjing Jiancheng Bioengineering Institute) following kit instructions, respectively. In short, to detect pyruvic acid, mix sample with extraction solution then incubate and centrifuge to collect supernatant. Preheat microplate reader to 520nm. Dilute 20μmol/mL standard stock 40× with extraction solution to obtain 0.5μmol/mL working standard. Preheat microplate reader to 520nm and add reagents as instructed. Read absorbance at 520nm immediately after 5min incubation at room temperature (25°C) and calculate PA content (μg/mL).

To detect lactic acid, the sample (collected or extracted), enzyme working solution and chromogenic agent were vortex-mixed and incubated at 37°C for 10 min. Add the terminator to stop the reaction and measure absorbance at 530nm. Align colorimetric measurements with concentration (mmol/L) through standard curve.

### ChIP analysis

We employed A commercial ChIP assay kit, (Millipore, Burlington, MA, USA) to validate the binding site of Hif1-α and the promoter region of Mettl3. Treated cell samples were harvested for ultrasonic treatment and centrifugation to yield supernatant containing DNA fragments for immunoprecipitation with Hif1-α (Cat#36169; Cell Signaling Technology, Danvers, MA, USA). Immunoprecipitated DNA fragments were then identified by PCR.

### RNA stability

Before adding 5 mg/mL actinomycin D (Sigma, SBR00013), transfect cells with nonsense control or Mettl3 siRNA. Then extract total RNA from cell samples at different time points for qRT-PCR to detect mRNA degradation.

### m6A dot blot

Extract RNA from cell samples and adjust RNA concentration as consistent. Mix 20X SSC Buffer and 37% formaldehyde (3:2) then add RNA of the same volume. Incubate the mixture at 95°C for 5 minutes to denaturate RNA. Drop 2μl treated RNA on NC membrane and thencrosslink under 302nm UV light. Quench membranes with PBST before 2-hour blocking with 5% skim milk. Incubate membranes with m6A antibody at 4°C overnight and with corresponding secondary antibody the next day at room temperature for 1 hour. After adequate washing with PBST, incubate membranes with ECL and then observe m6A level under chemiluminescence imaging system.

### Methyltransferase activity assay

MTase-Glo Methyltransferase Assay (V7601, Promega (Beijing) Biotech Co., Ltd) were used to detect the enzyme activity of Mettl3. Procedure was conducted strictly according to the instruction book. In short, mix 5 nM enzyme (Mettl3-Mettl14 complex, Active motif) and 200nM RNA substrate (5'P-uacacucgaucuggacuaaagcugcuc-3', synthesized by Genechem, Shanghai, China) and then add into a 96-well plate with Levosimendan diluted into different concentrations. Incubate this mixture at room temperature for 5-10 minutes after centrifugation (1000rpm, 2 min). Add 2.5μM SAM to initiate methyltransferase reaction, and incubate the mixture at 37°C for 30 minutes after centrifugation (1000rpm, 2 min). Add MTase-Glo™ Detection Solution and incubate the mixture at room temperature for 60 minutes. Measure the luminescence value using a plate reading luminometer.

### Statistical analysis

For comparison between two groups, two-tailed Student t tests were used, while for comparison among multiple groups, One-way ANOVA or Two-way ANOVA were used. Quantitative data were presented as mean ± SD. p < 0.05 was regarded as statistically significant.

## Supplementary Material

Supplementary figures.

## Abbreviations

BUMPT: Boston university mouse proximal tubule
AKI: acute kidney injury
CKD: chronic kidney disease
CDDP: cisplatin
Mettl3: Methyltransferase 3
BUN: blood urea nitrogen
