# Supplementary material for: Inhibition of Mettl3 alleviates low-dose cisplatin-induced renal fibrosis and enhances the chemotherapeutic efficacy in mouse models of cancer
Source: Int J Biol Sci. 2025 Jun 23;21(10):4293–311. doi: 10.7150/ijbs.117443 (PMC12320029; doi:10.7150/ijbs.117443)
Supplement: Supplementary file 1 — Supplementary figures. [file ijbsv21p4293s1.pdf]

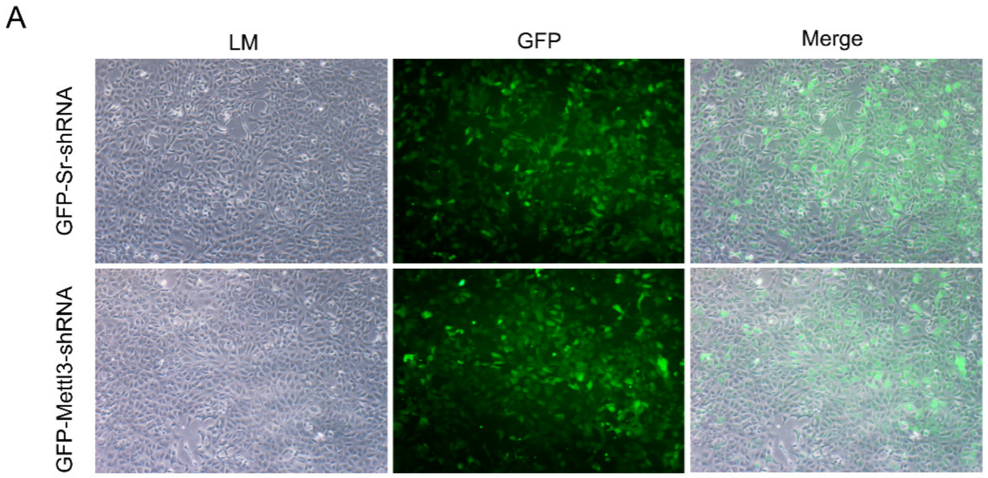

Figure S1. Establishment of a Mettl3 stable knockdown cell line A. Observation of the transfection efficiency of GFP-scramble (Sr)-shRNA and GFP-Mettl3-shRNA through fluorescence microscopy.

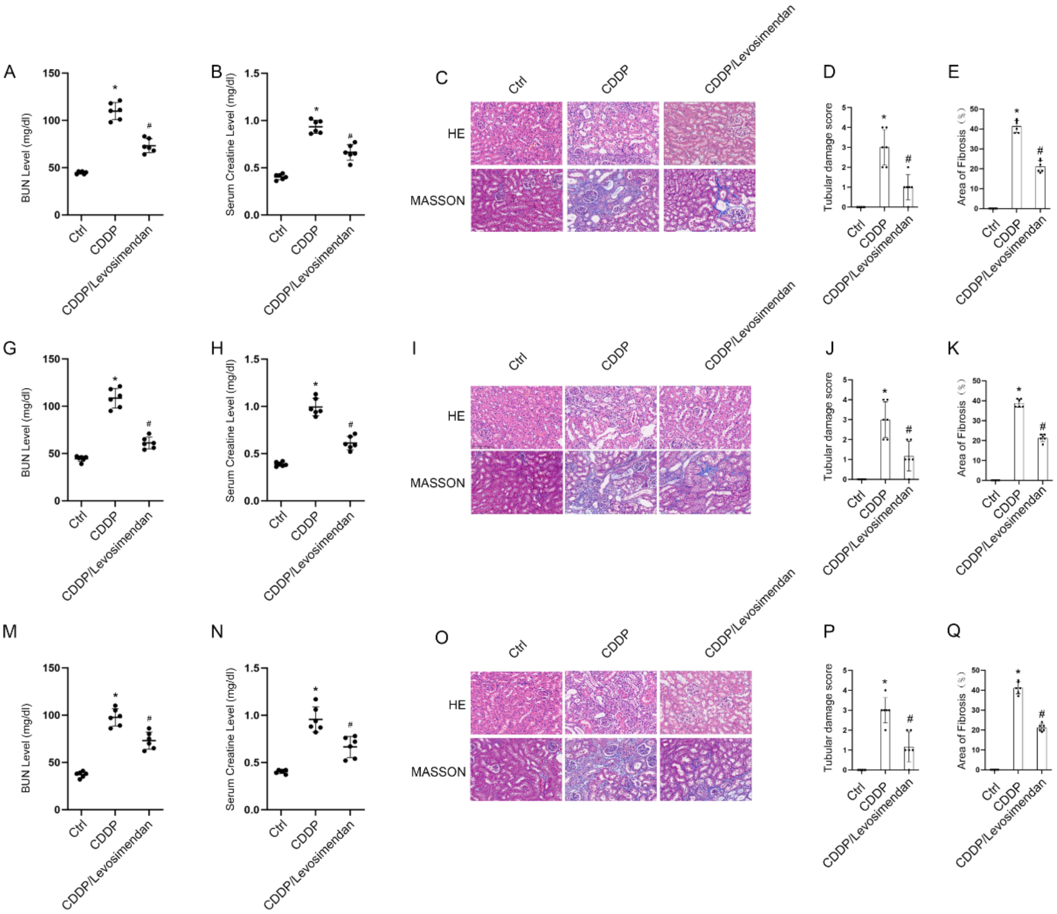

Figure S2. PLGA encapsulated-Levosimendan ameliorates CDDP-induced renal fibrosis.

Measurements of renal function indicators BUN and serum creatinine of nude mice enduring PLGA, PLGA/CDDP or PLGA-Levosimendan/CDDP with A2780 (A&B), MDM-231 (G&H) or T24 (M&N) tumor xenografts. C, I&O. H&E staining and Masson’s trichrome staining. D, J and P. Tubular damage score. E, K&Q. Area of fibrosis. *P < 0.05 versus ctrl. #0.05 versus CDDP.
